# Laparoscopic Ventral Hernia Repair Combined with Sleeve Gastrectomy in Morbidly Obese Patients: Early Outcomes

**DOI:** 10.1055/s-0039-1694979

**Published:** 2019-08-28

**Authors:** Ahmed M.S.M. Marzouk, Heba O.E. Ali

**Affiliations:** 1 Department of General and Laparoscopic Surgery, Faculty of Medicine, Cairo University, Cairo, Egypt; 2 Department of General and Laparoscopic Surgery, Altenagelvin Area Hospital, United Kingdom; 3 Department of Laparoscopic and Bariatric Surgery, New Medical Centre Hospital, Abu Dhabi, United Arab Emirates; 4 Department of Radiology, Faculty of Medicine, Cairo University, Cairo, Egypt; 5 Department of Radiology, Altenagelvin Area Hospital, Londonderry, United Kingdom

**Keywords:** hernia, laparoscopic ventral hernia repair, morbid obesity, sleeve gastrectomy

## Abstract

**Background** Morbid obesity is a serious chronic condition with, among other symptoms, increased intra-abdominal pressure and subsequent abdominal wall hernias. The optimal management of these manifestations is still controversial. The objective of this study was to assess the early postoperative outcomes of a surgical approach combining laparoscopic ventral hernia repair (LVHR) with sleeve gastrectomy in morbidly obese patients.

**Methods** In this retrospective study, we reviewed the files of patients who are obese with a primary ventral hernia of less than 10 cm in diameter who received simultaneous laparoscopic sleeve gastrectomy and LVHR at our institution between February 2016 and July 2018. LVHR was performed using an intraperitoneal only mesh. The individual mesh size was chosen based on the number and size of the defects. Clinical and radiological follow-ups were performed between 9 and 15 months.

**Results** A total of 15 patients were included. Five of them were males. The mean body mass index was 45.2 kg/m ^2^ (range: 38.7–56.2 kg/m ^2^ ). The mean hernia defect size was 2.6 cm (range: 1.3–4.2 cm). Mesh size was 10 × 15 cm in five, 20 × 15 cm in seven, and 25 cm× 20 cm in three patients. All patients were discharged without complications on the second postoperative day. Mean follow-up was at 12 months. One patient presented with hernia recurrence 14 months after surgery and four patients presented with self-limited seroma.

**Conclusion** Despite ambiguous guidelines and ongoing debate regarding simultaneous bariatric surgery and ventral hernia repair, the short-term outcomes of this approach appeared promising, provided that patients are carefully selected and receive an individually tailored approach.

Obesity is associated with and also a cause of numerous significant diseases. It is considered to be one of the most common risk factors for the development of anterior abdominal wall hernias. 1 This may be explained by the raised intra-abdominal pressure and reduced compliance of the abdominal wall associated with obesity. In addition, obesity is linked to poor wound healing. 2 The resulting risk of developing incisional and recurrent hernias is reflected in a continuous increase in the incidence of such hernias in patients who are obese. 3 4 5 6 Owing to the progressive character of obesity, the rate of abdominal wall hernia repairs in these patients increases proportionately. However, this poses the challenge of determining the optimum time and type of ventral hernia repair in view of high-recurrence rates. Therefore, surgical management of obesity is a valid option in those patients. 7

Ventral hernia repair is one of the most frequent interventions in general surgery including the repair of incisional and recurrent types. 8 Several techniques are described for both open and laparoscopic approaches without a significant difference in recurrence rates. However, the laparoscopic approach has the advantages of less wound-related complications, faster recovery, and rapid improvement of quality of life. 9 10 11 Many studies considered laparoscopic ventral hernia repair (LVHR) with intraperitoneal only mesh (IPOM) as a gold standard. 1

The indication for LVHR is based on a variety of factors that include the experience of the surgeon and the technology available. In addition, the characteristics of the hernia including the number and size of defects, the nature of its contents, loss of domain, and prior repair(s), which suggest the need for adhesiolysis, significant technical difficulty, and long operation times, must be duly considered. 7 8 9 10 11

Currently, the best approach in the management of patients who are candidates for weight loss or metabolic surgery and concomitantly have anterior abdominal wall hernias is still a matter of debate owing to the absence of an evidence-base for consensus. The main controversy is around whether to perform mesh repair in a clean-contaminated field with the risk of infection or anatomical mesh-free repair with its known increased rate of recurrence. At the same time, the postoperative risk of hernia incarceration or strangulation is a concern if it is left without repair. 12

The purpose of this study was to assess the early postoperative outcomes of simultaneous LVHR and sleeve gastrectomy in patients who are morbidly obese.

## Methods

This retrospective study was performed in the General, Laparoscopic, and Bariatric Surgery New Medical Centre Hospital in United Arab Emirates with institutional ethical committee approval. We reviewed the medical records of consecutive patients who are morbidly obese and who received laparoscopic sleeve gastrectomy (LSG) based on multidisciplinary selection combined with LVHR between February 2016 and July 2018.

Obesity is defined according to the body mass index (BMI), which is calculated as body weight divided by the square of body height. A BMI of 25–29.9 kg/m ^2^ is considered as overweight and a BMI > 30 kg/m ^2^ as obesity. Patients with a BMI > 40 kg/m ^2^ or a BMI > 35 kg/m ^2^ with comorbidities (hypertension, type-2 diabetes mellitus [T2DM], obstructive sleep apnea [OSA], and bronchial asthma) are considered morbidly obese. In this study, we included patients who were morbidly obese, had a primary ventral hernia, a total defect(s) size < 10 cm, and provided informed consent for their participation and follow-up.

We excluded patients with a loss of domain, a history of previously infected mesh grafts, and current skin changes over the hernia sac.

**Fig. 1:**
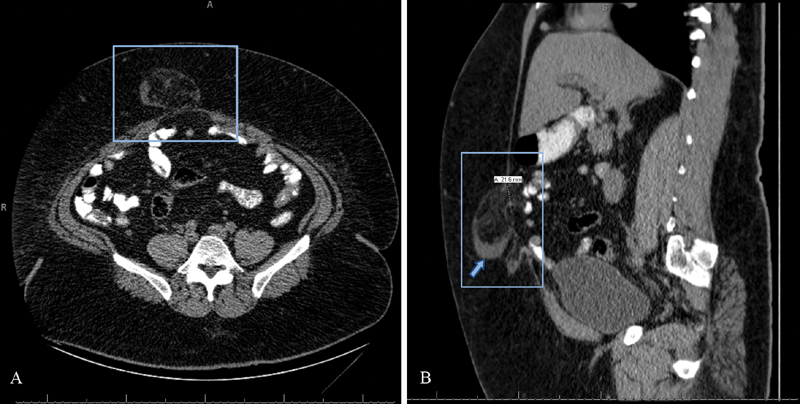
Preoperative axial ( **A** ) and sagittal ( **B** ) computed tomography assessment of midline defects in a male patient with BMI, 56.2 kg/m ^2^ . Supraumbilical hernia with defect measuring 21.6 mm with omentum as its content is seen. Subtle soft tissue thickening seen in the inferior aspect of hernia sac (arrowed). BMI, body mass index.

All patients had been evaluated with a preoperative computed tomography of the abdomen and pelvis for assessment of the defect size ( Fig. 1 ), contents of the hernia sac, and any associated pathologies.

**Fig. 2:**
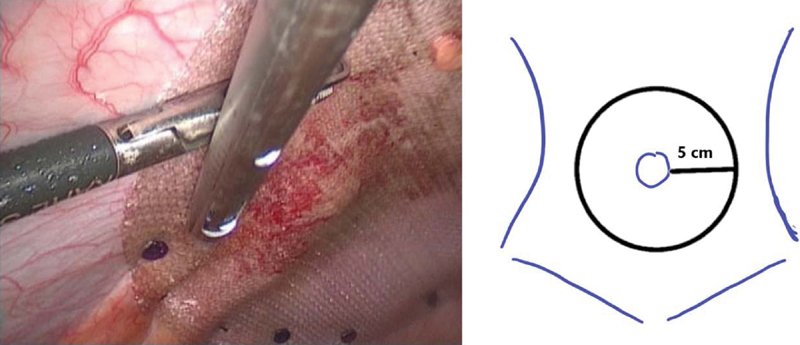
Intraoperative view: laparoscopic ventral hernia repair was performed using intraperitoneal onlay mesh covering an at least 5 cm margin around the defect(s). The composite mesh was fixed with an absorbable fixation device and transfascial sutures.

Our LSG technique uses a 36-Fr gastric calibration tube and starts approximately 2 to 4 cm from the pyloric ring. LVHR is performed thereafter, adopting the IPOM technique with a suitable composite synthetic mesh (three-dimensional monofilament polyester textile, with an absorbable, continuous, and hydrophilic film on one side). The size was determined based on the number and size of the defects and calculated based on a circumferential coverage of a margin of at least 5 cm around the defect(s) ( Fig. 2 ). We routinely use both transfascial sutures and absorbable tacks for mesh fixation. A routine ultrasound-guided transversus abdominis block (TAB) is administered as part of our enhanced recovery protocol.

All patients had postoperative pain assessment at every 6 hours using visual analog scale with a scale from 0 (no pain) to 10 (worst pain). A pain score of 2 to 3 out of 10 was one of our discharge criteria.

We compared the findings for operation time, pain score, and length of hospital stay with our institutional means. Clinical and radiological assessments were performed after 9 and 15 months. All collected data revised for completeness and accuracy with statistical analysis. Data were summarized using mean and standard deviation (SD) for quantitative variables and number and percent for qualitative variable. Comparison between qualitative variables was done using Chi-square. A *p* -value less than 0.05 was considered statistically significant.

## Results

The study population ( Table 1 ) consisted of 15 patients (10 female and 5 male). The mean BMI was 45.23 kg/m ^2^ (range: 38.7–56.2 kg/m ^2^ ). Seven patients (46.7%) had hypertension, six (40%) had T2DM, two had a mild form of OSA (no continuous positive airway pressure needed), and three (20%) had bronchial asthma.

**Table 1:** Patients' baseline characteristics ( *n* = 15) prior to combined hernia repair and bariatric surgery

Patients	
Sex	10 F/5 M
Age (y)	Mean 42.7 (range: 37–51)
BMI (kg/m ^2^ )	Mean 45.23 (38.7–56.2)
Comorbidities	
•Hypertension	7 (46.7%)
•Type-2 DM	6 (40%)
•OSA	2 (13.3%) mild
•Asthma	3 (20%)
– Number of defects	(1–3)
– Defect(s) size (cm)	Mean 2.63 (range: 1.3–4.2)

Abbreviations: BMI, body mass index; DM, diabetes mellitus; F, female; M, male; OSA, obstructive sleep apnea.

**Fig. 3:**
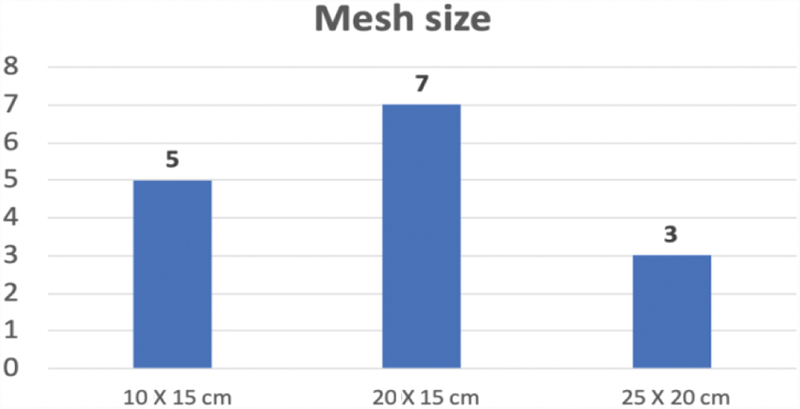
Bar chart showing different mesh sizes used for hernia repair according to defect size.

**Fig. 4:**
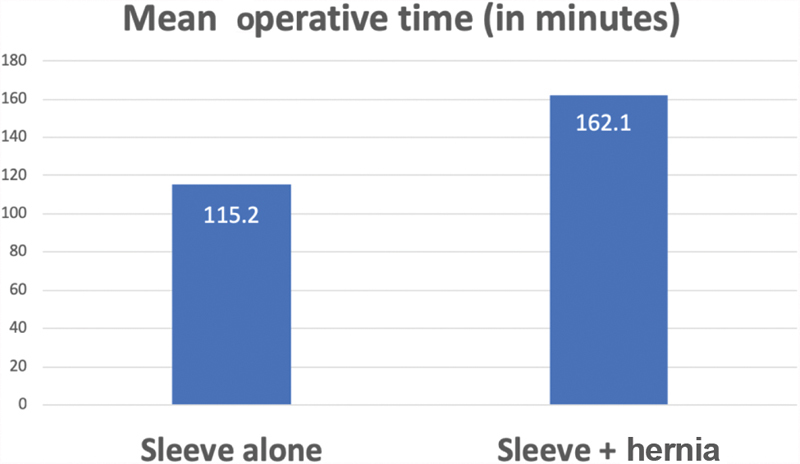
Comparison of mean operation time between laparoscopic sleeve gastrectomy only and sleeve gastrectomy combined with hernia repair.

The number of defects per patient ranged between one and three, and the mean defect size was 2.63 cm (range: 1.3–4.2 cm). Dependent on the individual size and number of defects, a 10 × 15 cm mesh was used in five patients, a 20 × 15 cm mesh in seven patients, and a 25 × 20 cm mesh in three patients ( Fig. 3 ). The mean operation time was 115.2 minutes in LSG only and 162.1 minutes in LVHR which was not statistically significant ( *p* > 0.05) ( Fig. 4 ).

There was no significant difference in the mean postoperative pain score (mean = 2.73) when compared with our institutional mean pain score for patients after LSG alone ( *p* > 0.05).

All patients were discharged on the second postoperative day and were followed for a mean of 12 months ( Table 2 ). One patient (6.6%) had hernia recurrence 14 months postsurgery, four patients (26.6%) had a self-limited superficial seroma with a mean resolution time of 5.5 weeks (range: 4–7 weeks). No other complications were reported. With regards to excess weight loss, there was no statistically significant difference in comparison to our institutional outcomes (49.4 versus 51.6 kg; *p* > 0.05).

**Table 2:** Follow-up outcomes in patients ( *n* = 15) after combined hernia repair and bariatric surgery

Follow-up outcomes	
Hernia recurrence	1/15 (6.6%)
Seroma formation	4/15 (26.6%)
Seroma resolution (wk)	4–7 (mean = 5.5)
Excess weight loss (kg)	61.7 ± 26.3 (mean = 49.4)

## Discussion

Patients who are morbidly obese are more likely to show abdominal wall extension and, hence, are predisposed to develop hernias, including their potential complications, such as small bowel obstruction as a common cause of morbidity and mortality. 13 Laparoscopic approaches are associated with a lower infection risk and a shorter hospital stay than open abdominal surgery. They also provide a better ability to identify occult hernias or weakening of the fascia, reducing operative failure and long-term recurrence. 7

Obesity is a key factor for incisional hernias. Therefore, many patients who are obese already received hernia repair before bariatric surgery. Modern hernia repair approaches and the continuously evolving techniques in minimally invasive surgery have increased the opportunities to treat patients in a one-stage procedure to reduce the risk of incisional hernias. 13 14

Previous findings suggest that weight loss prior to the operation may help to enhance the technical circumstances and reduce the recurrence rate, though it appears not to change the risk of perioperative complications. 15 However, realistic expectations and the time necessary to achieve significant weight loss need to be weighed against the clinical indication for both hernia repair and bariatric surgery. 13

Ching et al 15 reported a 12% recurrence rate after standard LVHR that is influenced by both the defect and mesh size. However, recurrence was not influenced by the degree of obesity. Schuster et al 16 reported two recurrences in 12 patients who received concurrent laparoscopic Roux-en-Y gastric bypass (LRYGB) and anterior wall hernia repair at the 14-month follow-up. All patients had prosthetic mesh repair of their hernias using either polypropylene and cellulose or polyester and collagen, except one who received primary repair. With regards to mesh infections, especially in a clean-contaminated field, Kaul et al showed a low infection and recurrence rate in their prosthetic mesh repairs. 17

According to recent guidelines, LVHR requires trained experienced laparoscopic surgeons, as well as the optimal resources, in terms of the availability of suitable mesh and fixation devices to achieve the best outcomes. 7 In our practice, we routinely combine transfascial fixation with fixation tacks with a mesh overlap of 3 to 5 cm to minimize recurrence. This strategy can decrease recurrence rates by 50% and more. 7

Despite the more complex procedure in comparison to sleeve gastrectomy only, the addition of hernia repair was not associated with a significant increase in postoperative pain or hospital stay. A possible explanation is our routine performance of TAB, but less postoperative pain and faster recovery remain the main advantages of laparoscopic repair over open approaches. In addition, the longer duration of the intervention does not significantly affect the outcomes. 9 10 11

Owing to the potential space between the bridging mesh and the covered defect, seroma formation is an acknowledged postoperative outcome. 18 In our study, we encountered a 26.6% incidence of seromas that were all self-limited and did not need intervention.

For patients with a ventral hernia requiring a bariatric procedure, the majority of the data suggests that a combined surgical approach is safe and should be considered. 12 13 14 It is important to note that the procedures are more commonly performed in conjunction with LVHR are LRYGB and laparoscopic adjustable gastric banding, whereas fewer studies provide data on LSG.

When considering our results, we acknowledge that this study has some limitations. Our study population was very small and larger and randomized comparative studies with longer follow-up periods are needed to accurately identify the pros and cons of simultaneous hernia and bariatric operations.

## Conclusion

In our experience, simultaneous performance of bariatric surgery and ventral hernia repair results in promising short-term outcomes provided that patients are carefully selected, and the approach is tailored to their individual situation.
